# The impact of the severity of Obstructive Sleep Apnea syndrome on quality of life

**DOI:** 10.1192/j.eurpsy.2024.986

**Published:** 2024-08-27

**Authors:** A. Fki, A. Ben lazreg, R. Bouchech, R. Ben Cheick, G. Sakly

**Affiliations:** ^1^occupational medicine department, Sahloul university hospital; ^2^Faculty of Medicine; ^3^department of Neurophsiology, Sahloul university hospital, Sousse, Tunisia

## Abstract

**Introduction:**

The negative impact of obstructive sleep apnea syndrome (OSA) on the quality of life of affected individuals is one of the serious consequences of this pathology. Consideration of this quality of life as one of the therapeutic objectives is essential.

**Objectives:**

to evaluate the impact of the severity of OSA on quality of life in affected patients

**Methods:**

We conducted a cross-sectional study involving 40 patients diagnosed with OSA by polysomnography in the Sleep unit, department of Neurophysilogy at Sahloul university hospital in Sousse, Tunisia. This study was based on a generic questionnaire (SF-12) to assess the quality of life.

**Results:**

The mean age was 49.7 ± 7.87 years with a sex ratio of 1.10. The mean apnea-hypopnea index (AHI) was 29.72. OSA was mild, moderate and severe in 40%, 22.5% and 37.5% of cases respectively. The majority of our patients had an impaired quality of life with an ave[1]rage score of 42.78. There was a positive linear relationship between physical and mental components of the SF-12 and AHI (p= 0.026 and p=0.019 respectively). Mental component of the SF-12 was significantly associated with treatment with CPAP (continuous positive airway pressure) (p=0.014).

**Conclusions:**

Our study has shown that the severity of OSA has an impact on different domains of quality of life. The management of this disease should not be limited to controlling the disease but should aim for overall patient satisfaction.

**Disclosure of Interest:**

None Declared

